# Cell-type specific pallial circuits shape categorical tuning responses in the crow telencephalon

**DOI:** 10.1038/s42003-022-03208-z

**Published:** 2022-03-25

**Authors:** Helen M. Ditz, Julia Fechner, Andreas Nieder

**Affiliations:** Animal Physiology Unit, Institute of Neurobiology, University of Tuebingen, Auf der Morgenstelle 28, 72076 Tuebingen, Germany

**Keywords:** Neural circuits, Cognitive neuroscience

## Abstract

The nidopallium caudolaterale (NCL), an integration centre in the telencephalon of birds, plays a crucial role in representing and maintaining abstract categories and concepts. However, the computational principles allowing pallial microcircuits consisting of excitatory and inhibitory neurons to shape the tuning to abstract categories remain elusive. Here we identified the major pallial cell types, putative excitatory projection cells and inhibitory interneurons, by characterizing the waveforms of action potentials recorded in crows performing a cognitively demanding numerical categorization task. Both cell types showed clear differences in their capacity to encode categorical information. Nearby and functionally coupled putative projection neurons generally exhibited similar tuning, whereas putative interneurons showed mainly opposite tuning. The results favour feedforward mechanisms for the shaping of categorical tuning in microcircuits of the NCL. Our findings help to decipher the workings of pallial microcircuits in birds during complex cognition and to compare them vis-a-vis neocortical processes in mammals.

## Abstract (web summary)

Neural recordings from the caudolateral nidopallium in crows during a numerosity task suggest there are two subsets of projection neurons and inhibitory interneurons involved in complex cognition.

## Introduction

Birds are vertebrates with a telencephalon lacking a cerebral cortex; they evolved radically different telencephalon structures from different territories of the embryonic pallium since they diverged from the mammalian lineage 320 million years ago^1^. While mammals evolved a six-layered neocortex from the embryonic dorsal pallium as a major integration center, birds developed nuclear integration centers out of the ventral pallium^2–4^. Interestingly, and despite these differences in anatomical origins, the avian ventral pallium and the mammalian neocortex converged on similar neuronal circuits, such as layers and columnar organizations in sensory pallial areas^5–9^. However, these circuits in birds engage entirely separate classes of excitatory and inhibitory pallial neurons that have no counterpart in the mammalian neocortex^10^.

Nevertheless, some birds show sophisticated cognitive behaviors^11^. Neurophysiological recordings in behaving birds, have identified the pallial telencephalic structure “nidopallium caudolaterale (NCL)” as a key brain area representing complex cognitive capabilities^12–15^. Studies in behaving birds have identified neurons that encode stimulus association^16,17^, working-memory information^18^, motor plans^19^ in crows, and reward in pigeons^20^. In addition, NCL neurons are also involved in abstract categorization; they represent the number of items in a set, its numerosity. More precisely, NCL neurons are selectively tuned to preferred numerosities and show peak-tuning functions with a maximum response to an individual preferred numerosity^21–25^. To increase discrimination precision and to avoid extensive overlapping tuning curves, sharp neuronal tuning is required. However, how tuning functions to abstract categories are shaped by neuronal computations in the avian pallium remains elusive.

We hypothesized that local microcircuits consisting of projection neurons and interneurons play a crucial role in shaping tuning. These two major neuron types are not only comprising the mammalian neocortex^26–28^ but also the avian pallial alternative telencephalon structures^29–33^. About three-quarters of pallial cells are projection neurons (termed “pyramidal cells” in the mammalian neocortex) that are all excitatory, whereas the remaining one-quarter consists of mainly inhibitory and locally operating interneurons. These categorical cell classes show distinct electrophysiological properties that can be used for identification on the basis of extracellular recordings.

Using a combination of techniques, these physiological differences of pyramidal cells and interneurons have firmly been established for the neocortex^34,35^. The most important electrophysiological characteristic is that neocortical pyramidal cells typically show longer action-potential waveforms than interneurons^26,36–38^. Thus, putative pyramidal cells are referred to as broad-spiking neurons (BS), whereas putative interneurons are termed narrow-spiking neurons (NS). This correspondence between BS and pyramidal cells on the one hand, and NS and interneurons on the other, is particularly well established for the prefrontal cortex^35,39–41^, the mammalian analog of the avian NCL. The very same relationships have been reported for neurons in song nuclei of songbirds. Studies in which electrophysiology and histology were combined to identify the types of recorded neurons found that narrow waveforms of action potentials are derived from interneurons, whereas broad and long waveforms of spikes relate to projection neurons^29,30^.

In the current study, we used this well-established approach for discriminating these two cell classes based on extracellular recordings from crows discriminating numerosity. For a fraction of the neurons, we could establish functional connectivity by analyzing temporal spiking correlations. We show that putative pyramidal cells and interneurons in the avian nidopallium showed specific response properties and functional interactions, implicating distinct roles in shaping categorical representations. Our data provide evidence for local feedforward-inhibition processes, leading to a refinement of response tuning necessary for precise distinction between abstract categories.

## Results

### Classification of narrow- and broad-spiking neurons

**Fig. 1:**
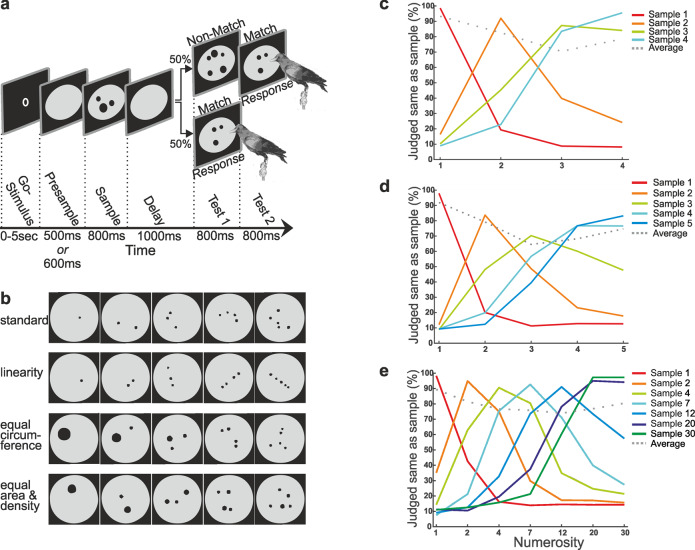
Delayed match to numerosity task and behavioral performance. **a** Behavioral protocol. The crows had to move their heads into an infrared light barrier when the go-stimulus appeared to start a trail. The first display contained 1–4, 1–5 (small numerosities) or 1–30 items (large numerosities) and was shown as 800 ms. The crows had to memorize this numerosity for 1000 ms over the delay period and match it to the subsequent test display. In 50% of the cases, the test contained the same number of items as the sample (match). By pecking on the screen, the crows indicated the match and received a reward. In nonmatch trials, the crows had to refrain from pecking on the screen until a second display appeared, which was always a match. **b** A small subset of the stimulus displays are shown as examples. The physical appearance of the displays varied widely for the same quantities for the numerosity set 1–5. **c, d, e** Behavioral performance in experiments with four (**c**), five (**d**), and 30 numerosities (**e**). The colors of the curves represent performance curves for a given sample numerosity. The average performance for each numerosity is shown in gray as the percentage correct (chance level, 50%).

We recorded the activity of single neurons in the nidopallium caudolaterale (NCL) of three crows engaged in a delayed match-to-numerosity task. The crows saw a number of dots they had to memorize over a brief delay period and match to the correct test numerosity in the test period (Fig. 1a). A maximum of recording data were compiled for the current study from projects in which numerosity from 1 to 4^23^, 1 to 5^22^, and 1 to 30^21^ were presented to the crows (Table 1). Low-level visual features of the dot display, such as the arrangement, the total area and density of the dots, were controlled for to ensure that the crows were using the numerical value of the dot arrays to solve the task (Fig. 1b). All numerosities were presented in each session with one standard and one control condition. For each session, many different dot displays per numerosity were generated with pseudorandomly varied visual properties. Moreover, sample and test stimuli were never identical to prevent the crows from pattern matching (see “Methods”). All crows performed the task 80–90% correctly and showed the characteristic approximate numerical-discrimination functions (Fig. 1c)^21–23^.

**Table 1:** Details on the data set.

Subject	Number of neurons	Studies	Number of sessions
Crow 1 (f)	226	Nieder & Ditz (2015)	39
	–	Nieder & Ditz (2020)	–
	69	Nieder & Ditz (2016)	21
Crow 2 (m)	260	Nieder & Ditz (2015)	42
	225	Nieder & Ditz (2020)	76
	269	Nieder & Ditz (2016)	41
Crow 3 (m)	–	Nieder & Ditz (2015)	–
	87	Nieder & Ditz (2020)	50
	–	Nieder & Ditz (2016)	–

**Fig. 2:**
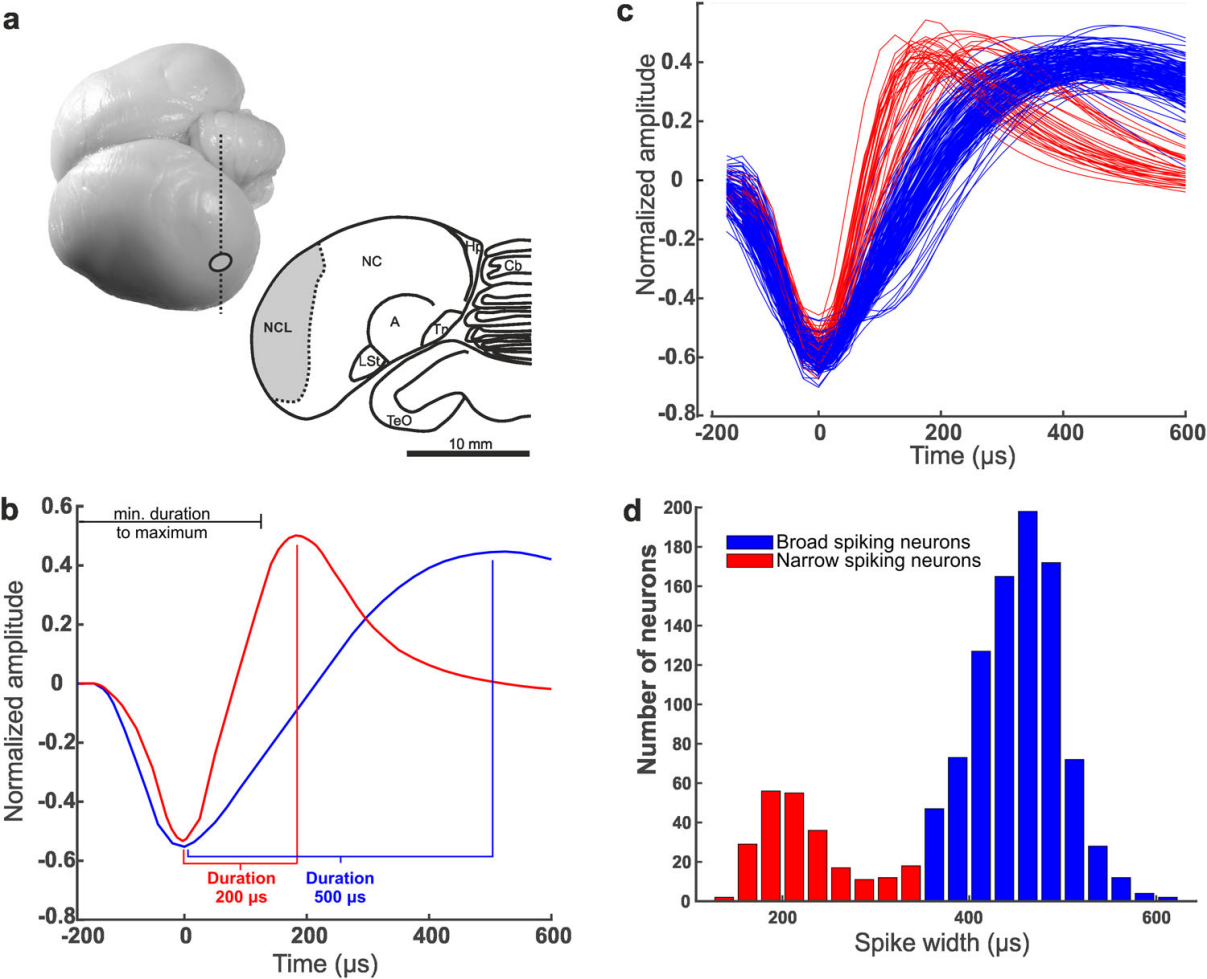
Recording site and classification of broad- and narrow-spiking neurons. **a** Recording sites in the crow NCL. Dorsolateral view of a carrion-crow brain. Vertical dashed line indicates section level (A5.00) and implantation site (circle). Coronal section (level A5.00) through the brain of a carrion crow illustrating the extent of the nidopallium caudolaterale (NCL) in the caudal telencephalon. **b** Mean waveforms of one narrow (red) and one broad (blue) spiking neuron recorded from the same electrode. The waveform duration is defined as the time from waveform valley to peak and was a characteristic used to classify neurons as broad- or narrow-spiking neurons. Only waveforms that showed a maximum no earlier than 300 µs after the initial amplitude threshold was reached were included. **c** Normalized average waveforms of a random subset of 300 neurons aligned by their minimum. Waveforms of narrow-spiking neurons are depicted in red; waveforms of broad-spiking neurons are depicted in blue. **d** Bimodal distribution of waveform durations.

Spiking activity of a total of 1169 extracellularly recorded single neurons in the nidopallium caudolaterale (NCL) (Fig. 2a) was sorted based on waveform characteristics using principle components and other characteristic waveform parameters such as times and amplitudes of minimum and maximum of the waveform. Because the largest-amplitude deflection is expected downward in extracellular recordings without signal inversion, the amplitude threshold for spike detection was set below baseline. All NCL neurons (1136/1169) with a downward voltage deflection followed by an upward voltage deflection with a clear peak in their waveforms were analyzed (Fig. 2b). The remaining 33 NCL neurons with deviating waveforms were excluded from further analysis (see Methods for details of exclusion criteria).

We hypothesized to find two separable distributions of waveforms corresponding to putative inhibitory interneurons (narrow-spiking) and putative excitatory projection neurons (broad-spiking)^32,42^. To test for a bimodal distribution, we calculated the average action-potential waveform for the 1136 NCL neurons. The neurons’ action potentials had comparable biphasic shapes but varied in duration, as defined by the interval between the waveforms’ troughs and peaks (Fig. 2c). Waveform durations exhibited a significant bimodal distribution (*p* < 0.001; Hartigan’s and Hartigan’s dip test for unimodality^43^, with the two modes of the bimodal distribution corresponding to the classification results) (Fig. 2c). The waveform durations were objectively segregated into narrow- and broad-spiking neurons by a linear classifier (Fig. 2c). Waveform classification resulted in 19% (218/1136) narrow waveforms with a peak distribution at ~200 ms, and 81% (918/1136) broad waveforms with a peak distribution at ~500 ms duration (Fig. 2d). We use the descriptive terms narrow-spiking neurons (NS) and broad-spiking neurons (BS) in the following to denote putative interneurons and projection neurons, respectively.

### General physiological characterization of narrow- and broad-spiking neurons

**Fig. 3:**
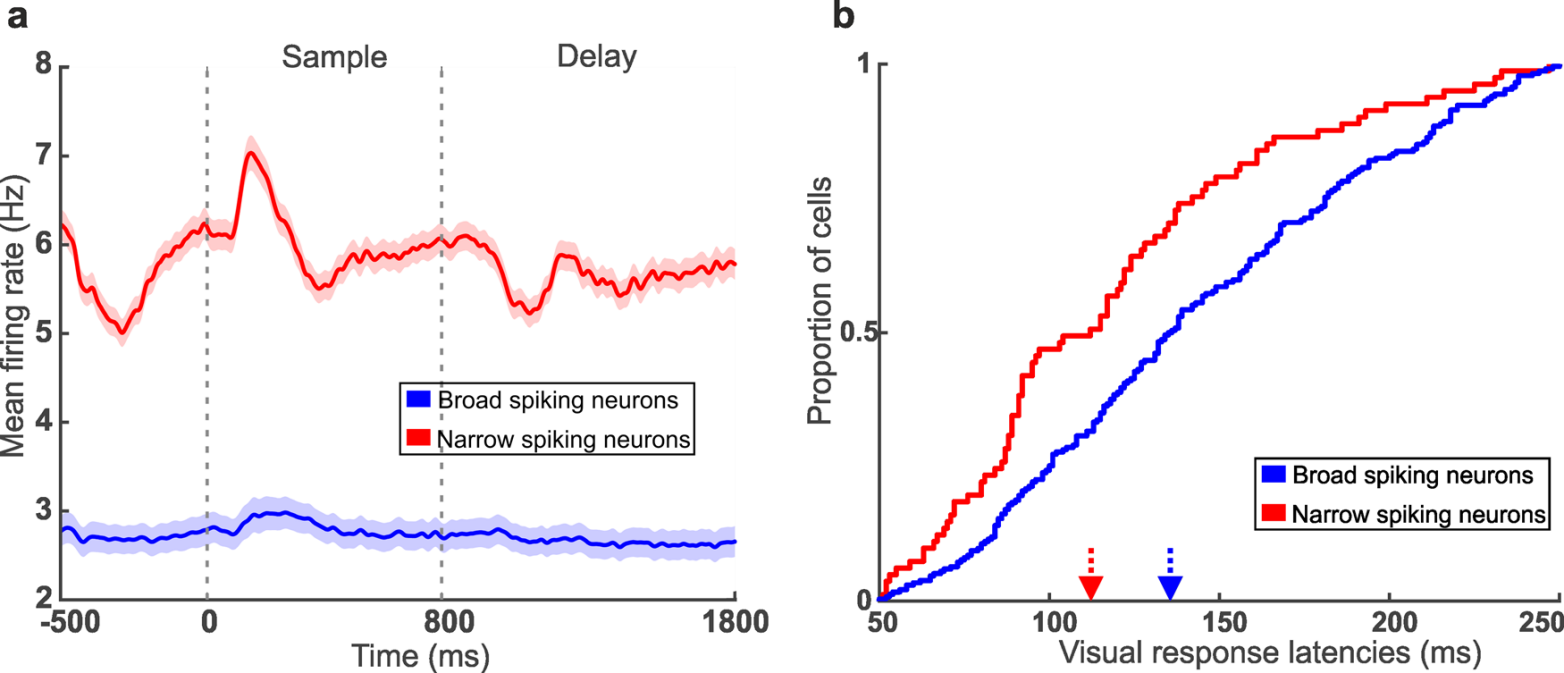
Basic physiological differences of NS and BS. **a** Mean firing rates during trial phases for NS (red) and BS (blue) neurons. **b** Cumulative histogram for response latencies. Arrows indicate median latencies for NS (red) and BS (blue) neurons.

After classification of neurons into BS and NS, we compared basic physiological properties of both neuron types. We found that, first, the rising slope of the spike waveform, defined as the difference between the amplitude of the peak and the trough divided by the time difference of the peak and the trough, was smaller for BS (median_BS_ = 0.06) than for NS (median_NS_ = 0.13) (Mann–Whitney U-test, U = 421821, *n*_BS_ = 918, and *n*_NS_ = 218, *p* < 0.001, two-tailed). Second, the average baseline firing rate of NS (median_NS_ = 3.82) Hz was higher than the average baseline firing rate of BS (median_BS_ = 1.83 Hz) (Mann–Whitney U-test, U = 157482, *n*_BS_ = 918, and *n*_NS_ = 218, *p* < 0.001, two-tailed) (Fig. 3a). Third, the visual response latencies of NS (median 112.0 ms) were shorter than those of BS (median 135.5 ms) (Mann-Whitney U test, U = 10114, *n*_BS_ = 918, *n*_NS_ = 218, *p* < 0.001, two-tailed) (Fig. 3b). Fourth, NS displayed strong ramping activity prior to stimulus onset, an effect absent for BS. Fifth, NS showed significantly stronger stimulus-evoked responses (median_NS_ = 4.30 Hz) than BS (median_BS_ = 1.88 Hz) during sample and delay periods (Mann–Whitney U-test, U = 165312, *n*_BS_ = 918, and *n*_NS_ = 218, *p* < 0.001, two-tailed). Seventh, the frequencies of BS and NS differed. Overall, 81% of the recorded NCL neurons were BS, whereas only 19% belonged to the NS (*χ*^2^ test, two-sided, *p* < 0.001). In summary, the two groups of NS and BS showed distinct physiological properties.

### Category-related response modulation of NS and BS cells

**Fig. 4:**
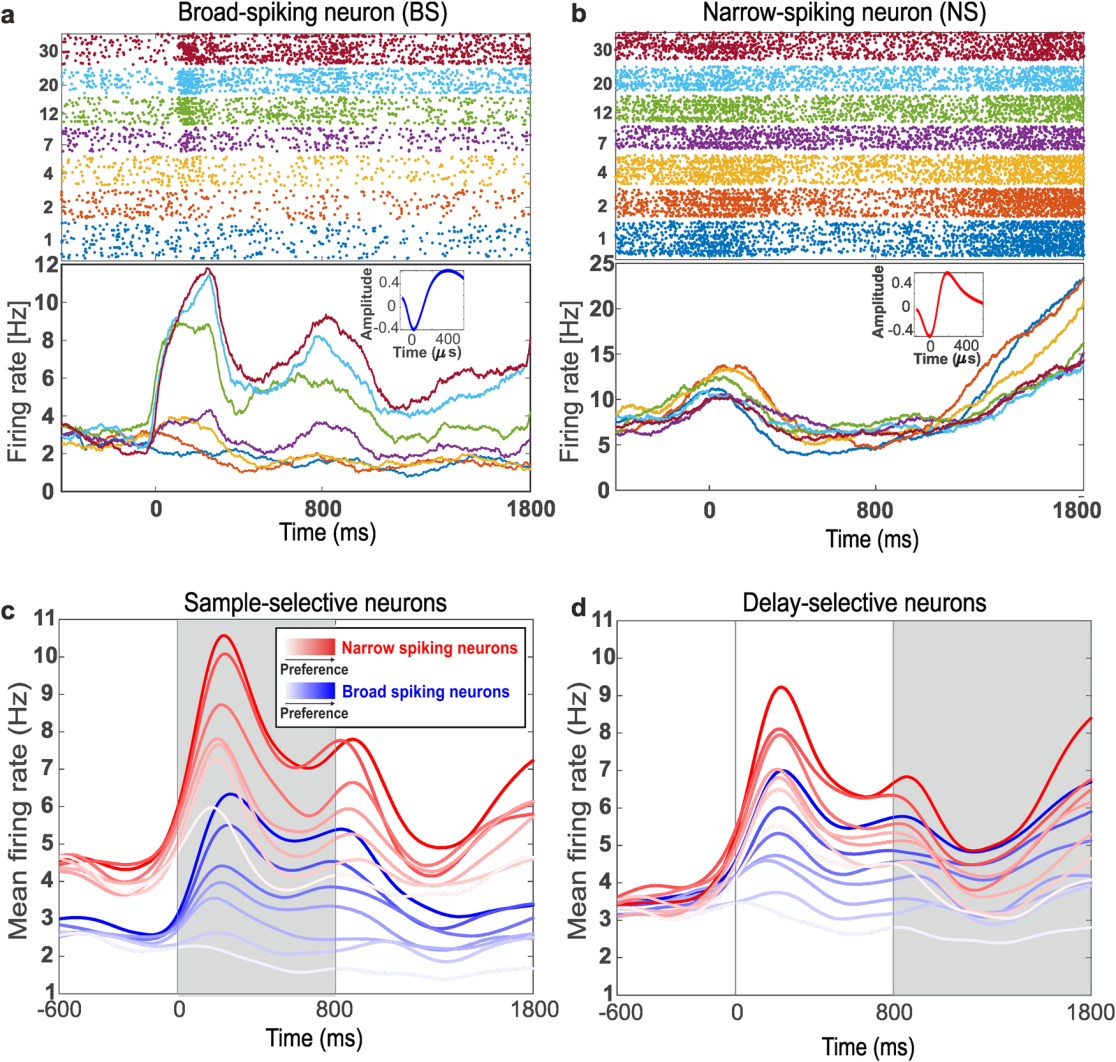
Firing-rate modulation of BS and NS numerosity-selective neurons. **a** Responses of an example BS neurons selective to numerosity 30 during the sample and delay periods. *Top panel*: dot-raster histograms with each dot representing one action potential. *Bottom panel*: averaged spike density function (activity averaged and smoothed by a 150-ms Gauss kernel). Colors in the dot-raster and spike density functions correspond the shown numerosity during the sample period. *Inset:* Average action potential waveform of this neuron. **b** Example NS neurons selective to numerosity 2. Same layout as in **a**. (**c,d**) Average spike-density histograms of BS and NS neurons that were selective for numerosities 1–30 during the sample (**c**) and delay (**d**) periods. Responses to more preferred (dark colors) and less preferred (light colors) numerosities are depicted.

Many neurons in the NCL selectively respond to quantity categories. They show peaked tuning curves with maximum discharge at their respective preferred numerosities. The detailed responses of single numerosity-selective neurons have been published^21–24^. We used a two-factor ANOVA (with factors ‘numerosity’ x ‘stimulus condition’ [standard vs. control]) to identify numerosity-selective neurons with only a significant main effect in numerosity (p < 0.01), but no other main or interaction effect, separately for sample or delay period. A total of 30% of the neurons (334/1136) were found to be numerosity-selective. Two example neurons are displayed in Fig. 4a, b. With respect to the task periods, 18% (202/1136) of the neurons were tuned during the sample period, 17% (195/1136) during the delay period. (These counts include 6% (63/1136) numerosity-selective cells during both sample and delay periods.). All further analyses are restricted to the 334 numerosity-selective neurons.

Next, we determined how many of the numerosity-selective neurons belonged to the NS and BS classes (Table 2). During the sample period, we found 22% (48/218) numerosity-selective NS (Fig. 4b) and 17% (154/918) numerosity-selective BS (Fig. 4a). During the delay period, 20% (44/218) NS and 16% (151/918) BS were found. These proportions of cell types among the numerosity-selective neurons were comparable in both task phases (*χ*^2^-test, two-sided, both *p* > 0.05).

**Table 2:** Frequency of broad- and narrow-spiking neurons and numerosity selectivity in different cell classes.

		Sample		Delay		Average (%)
Neuron class	*n*	Numerosity selective	%	Numerosity selective	%	
Broad spiking	918	154	17	151	16	17
Narrow spiking	218	48	22	44	20	21

We then compared the contributions of NS and BS to numerical categorization. To that aim, we ranked the neuronal responses of individual numerosity-selective neurons according to their numerosity preference and plotted the time-resolved discharges as average spike-density histograms separately for NS and BS (Fig. 4d, c). Because the data of the three different numerosity protocols could not be pooled for this analysis, only the data set recorded with the broad range of numerosities (1–30) is displayed. The responses of NS and BS are shown in red and blue in Fig. 4c, d, respectively, with increasingly fading color intensities from the respective preferred to the least-preferred numerosity (determined for each neuron separately prior to pooling).

We first analyzed neurons that showed numerosity selectivity to the broad range of numerosities (1–30) during the sample period (Fig. 4c). Both NS and BS showed elevated stimulus-evoked firing rates (NS: mean baseline-discharge rate: 4.2 ± 0.3 Hz; mean stimulus-evoked discharge rate during the first 250 ms of sample period: 7.7 ± 0.5 Hz; signed-rank test, *p* < 0.001; BS: mean baseline-discharge rate: 2.5 ± 0.1 Hz; mean stimulus-evoked discharge rate: 3.7 ± 0.2 Hz; signed-rank test, *p* < 0.001) during the first 250 ms of the sample period that specifically covers the phasic-response component. This elevation of the firing rates persists until the late sample phase (last 400 ms that specifically covers the tonic-response component) for both NS and BS neurons (NS: mean baseline-discharge rate: 4.2 ± 0.3 Hz; mean discharge rate during the last 400 ms of sample period: 5.5 ± 0.3 Hz; signed-rank test, *p* < 0.001; BS: mean baseline-discharge rate: 2.5 ± 0.1 Hz; mean discharge rate during the last 400 ms of sample period: 3.3 ± 0.2 Hz; signed-rank test, *p* < 0.001).

A similar picture emerged when we analyzed neurons that showed numerosity selectivity to the broad range of numerosities (1–30) during the delay period (Fig. 4d). During the early delay period (first 300 ms of the delay period), the firing rates of BS and NS to the preferred stimuli increased significantly compared with baseline activity (BS: mean baseline-discharge rate = 3.5 ± 0.2 Hz; mean discharge rate during the first 300 ms of delay period = 5.0 ± 0.2 Hz; signed-rank test, *p*_BS_ < 0.001; NS: mean baseline-discharge rate = 3.8 ± 0.2 Hz; mean discharge rate during the first 300 ms of delay period = 5.8 ± 0.3 Hz; signed-rank test, p_NS_ < 0.001, p_BS_ < 0.001). In a later delay phase (400 ms after delay onset, lasting for 400 ms), this significant elevation to the preferred numerosities persisted (most preferred stimuli: mean baseline-discharge rate = 3.8 ± 0.3 Hz for NS and 3.5 Hz ± 0.3 for BS; mean discharge rate during the last 400 ms of delay = 4.9 ± 0.3 Hz for NS and 4.8 ± 0.2 Hz for BS; signed-rank test, *p*_NS_ < 0.001, *p*_BS_ < 0.001).

### Selectivity of numerosity encoding for NS and BS cells

**Fig. 5:**
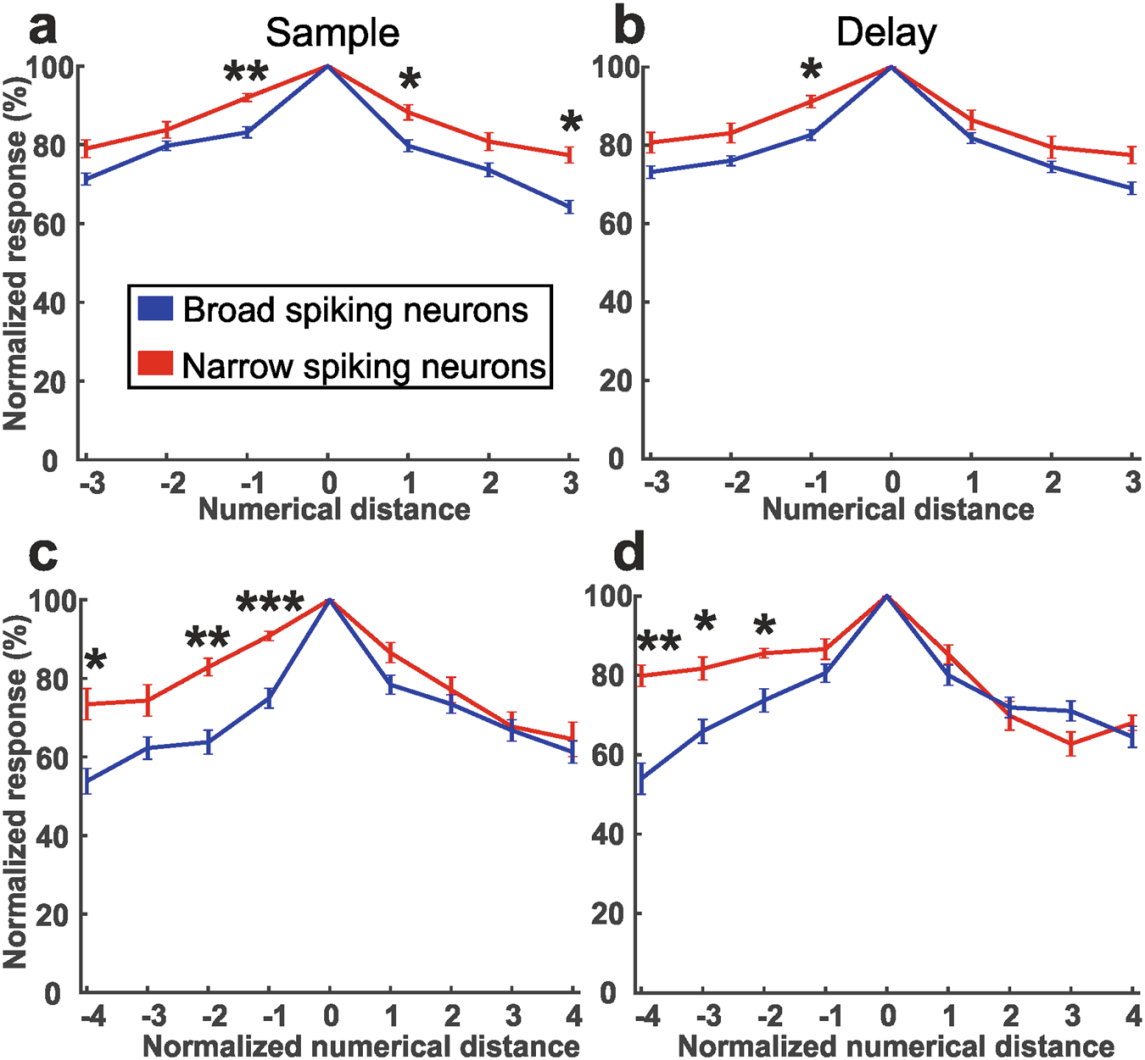
Population-tuning curves of NS (red) and BS (blue) neurons. **a, c** Population-tuning curves of neurons that were numerosity-selective during the sample period and that responded to small (1–4 and 1–5 pooled) numerosities (**a**) or large (1–30) numerosities (**c**). **b, d** Population-tuning curves of neurons tuned in the delay period and that responded to small numerosities (**b**) or large numerosities (**d**). For large numerosities, the distances between the tested numerosities are treated as having distance 1. Error bars indicate SEM. *p < 0.05, **p < 0.01, ***p < 0.001.

To explore putative category-selectivity differences between NS and BS, we calculated average tuning curves (with numerical distance as dependent variable). We found that NS exhibits broader tuning curves than BS in the sample (Fig. 5a) and delay phases (Fig. 5b) tested with small (1–4/5) numerosities. The same effect was found in the sample (Fig. 5c) and delay (Fig. 5d) for NS and BS tested with the broad range of numerosities (1–30). Asterisks displayed in Fig. 5 indicated the numerical distances for which responses of NS and BS differed significantly (*p* < 0.05, Mann–Whitney U test).

### Tuning properties of adjacent NS and BS cells

Under the assumption that NS and BS cells comprise different elements of microcircuits operating with inhibition and excitation, differences in category tuning for these cell types are expected. To find out, we explored the response properties of single cells recorded simultaneously at the same electrode tip. Such adjacent neurons are expected to interact more frequently than neurons recorded at more remote sites.

**Fig. 6:**
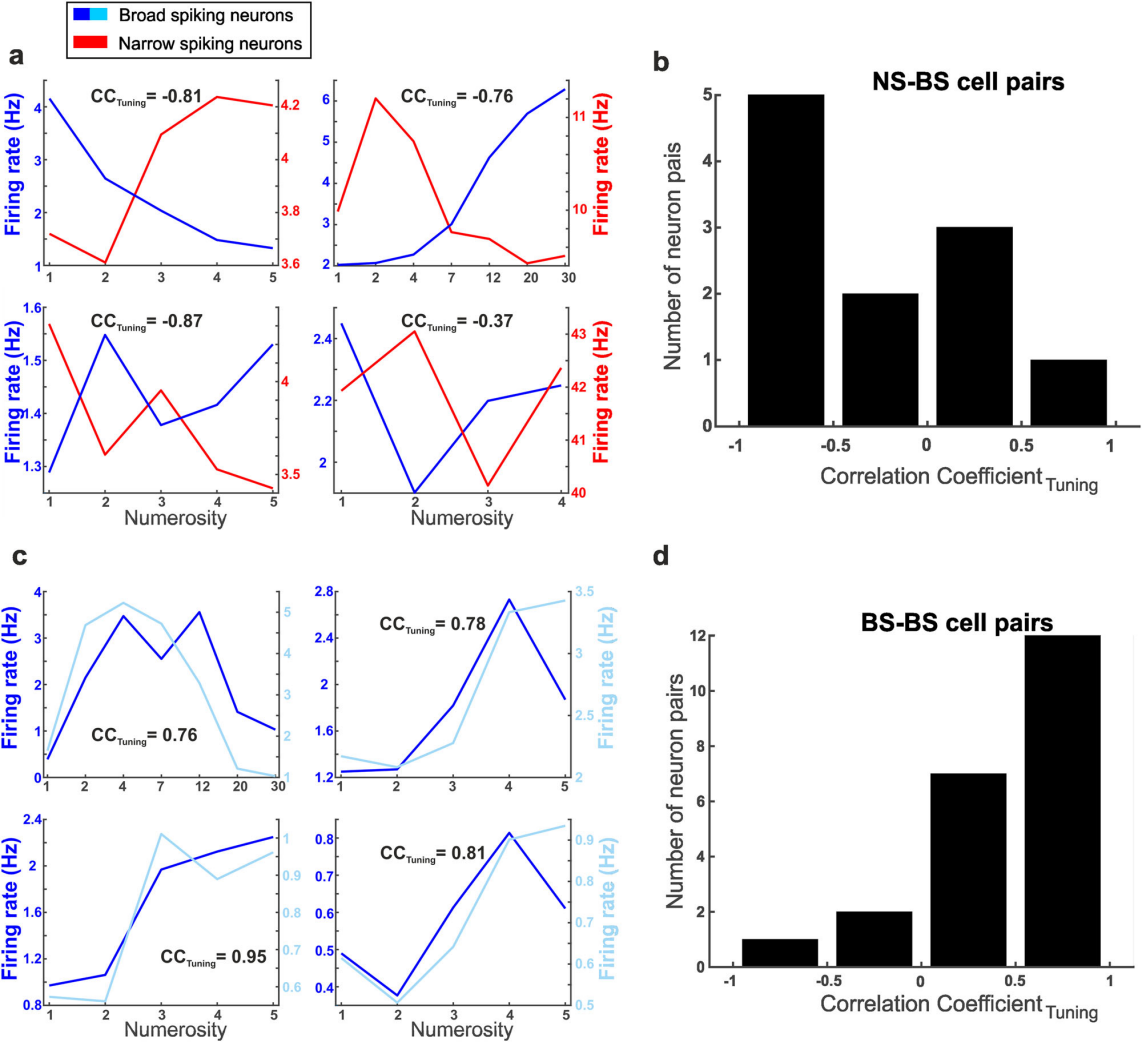
Tuning properties of adjacent numerosity-selective neurons. Tuning curves of four exemplary NS–BS (**a**) and four BS–BS neuron pairs (**c**) recorded at the same electrode. In (**a**), the tuning curves of BS cells are depicted in blue and the curves of NS cells in red. In **c**, the tuning curve of one BS neuron is shown in dark blue and the neighboring BS neuron in cyan. The CC_Tuning_ values represent the correlation coefficient of tuning curves. Note the different y axes for different cell pairs. **b, d** Histograms of CC_Tuning_ values of all pairs of numerosity-encoding NS and BS cells (**b**) and BS–BS cells recorded at the same electrode (**d**).

Indeed, numerosity-selective NS and BS simultaneously recorded at the same electrode often showed strikingly different tuning. Figure 6a depicts four exemplary NS–BS pairs. The neurons of each pair show more or less opposite tuning profiles: when one neuron is excited (to the preferred numerosity), the other is suppressed, and vice versa. To quantify this observation, we calculated the cross-correlation coefficient of tuning profiles (CC_Tuning_) as a measure of similarity and dissimilarity of tuning between cells (see Methods). A CC_Tuning_ of 1 would indicate a perfect match of tuning profiles, whereas a CC_Tuning_ of −1 would signify an exact inversion of the tuning curve of one neuron versus the other. For NS–BS cell pairs, a clear bias toward negative CC_Tuning_ values was observed (mean CC_Tuning_ value = −0.31; *n* = 11) (Fig. 6b). This argues that NS and BS in close vicinity have opposite tuning.

In contrast, BS–BS pairs (and the very few NS–NS pairs) showed highly similar tuning preferences. Figure 6c displays four example BS–BS pairs and their superimposed tuning curves. The similar tuning of BS–BS pairs resulted in highly positive CC_Tuning_ values (mean CC_Tuning_ value = 0.47; *n* = 22) (Fig. 6d). These tuning differences between NS–BS pairs and BS–BS pairs were significant: more NS–BS cell pairs than BS–BS pairs with a CC_Tuning_ smaller than −0.5 were found (Fisher’s exact test, *p* < 0.01; *n*_NS-BS_ = 9, *n*_BS-BS_ = 14). Inverted tuning was more frequent between adjacent NS and BS than between two neighboring BS cells. Finally, also the few NS–NS pairs showed highly positive CC_Tuning_ values (mean CC_Tuning_ value = 0.71, *n* = 4).

### Temporal interactions between cell pairs indicating functional connectivity

The analysis to this point is ignorant to the question whether neurons recorded at the same electrode tip might be functionally connected as parts of a microcircuit. To test whether and how (by excitation or inhibition) neuron pairs are functionally connected, we performed a temporal cross correlation analysis on spike timing and derived the cross-correlation coefficients (CC_Timing_) between neuron pairs recorded simultaneously at the same electrode (see Methods). If two neurons are functionally connected and one cell provides inhibitory input to the other, synchronous spiking should be suppressed, resulting in a negative correlation in the cross-correlogram at zero time lag. In contrast, if two neurons are functionally connected by excitation, synchronous spiking should be enhanced, resulting in a positive correlation in the cross-correlogram around zero time lag. These modes of functional connectivity can then be related to the tuning curves of cell pairs.

**Fig. 7:**
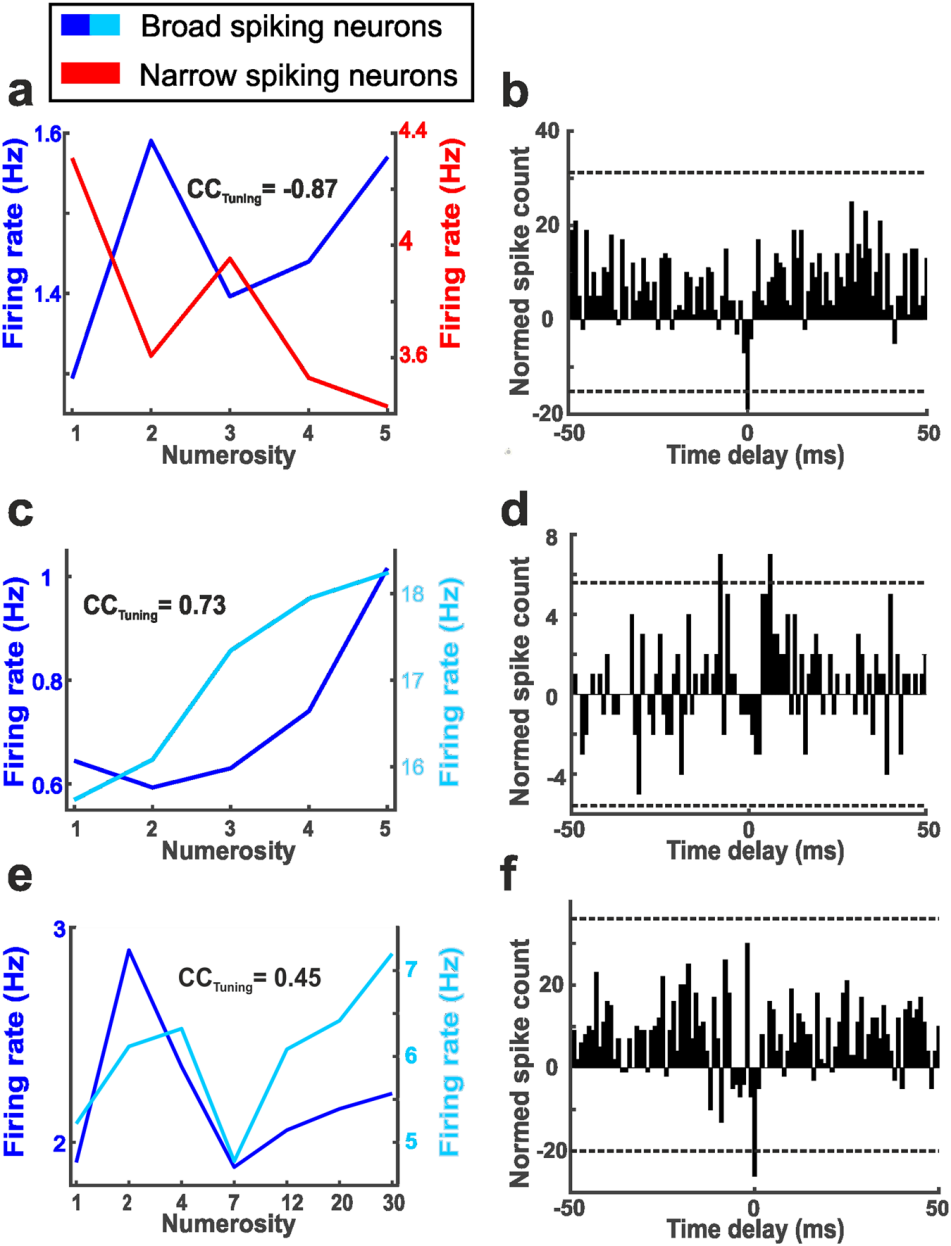
Synchronization pattern of adjacent numerosity-selective neurons. **a** Cell pair consisting of numerosity-tuned and temporally correlated NS and BS cells. The numerosity-tuning function of the respective NS and BS is displayed in **a**, the respective cross-correlogram (shift-corrected and baseline-subtracted; dotted lines indicate positive and negative significance thresholds, respectively) with a significant negative trough is shown in **b**. **c, d** and **e, f** show numerosity-tuned and temporally-correlated pairs of BS cells, same layout as in **a**.

We calculated the CC_Timing_ for the 24-numerosity neuron pairs that were tuned to numerosity in the same cognitive phase (sample or delay). This analysis could be performed with 7 NS–BS pairs, 16 BS–BS pairs, and one NS–NS pair. Of the 7 NS–BS pairs, 2 pairs showed significant CC_Timing_ values with a negative deflection in the cross-correlogram around zero time lag in addition to highly negative tuning (CC_Tuning_ < −0.5). The tuning functions of the NS and BS cell, which are inherently inverted relative to each other, and the cross-correlogram of one of these NS–BS pairs, are depicted in Fig. 7a, b.

For BS–BS cell pairs, a different picture of functional interactions emerged. Of the 16 BS–BS pairs, 7 showed significant CC_Timing_ values and 8 pairs showed significant positive CC_Tuning_ values. Four pairs were significant to both parameters. Of those, two pairs with a positive CC_Timing_ value showed also positively correlated tuning (CC_Tuning_ of 0.73 and 0.81). One of these BS–BS pairs is shown in Fig. 7c, d, in addition to a BS–BS pair that exhibits a positive CC_Tuning_ value and strong positive and negative deflections around zero time lag displayed in Fig. 7e, f. Finally, the only significantly temporally correlated NS–NS pair showed positive correlation in the cross-correlogram and positive correlation of the tuning functions (CC_Tuning_ = 0.56).

In summary, NS–BS pairs were mainly characterized by inverse numerosity tuning and negative cross-correlation effects. This argues that the inverse tuning is caused by inhibitory interactions between functionally connected NS and BS. In contrast, BS–BS exhibited mainly positive temporal correlation of spike trains and positively correlated tuning functions. This suggests excitatory connections causing spiking synchronization in neighboring BS cells.

## Discussion

In the current study, we report that extracellularly recorded neurons of the NCL of behaving crows can be classified into broad-spiking (BS) and narrow-spiking (NS) neurons based on their action-potential profile. These BS and NS show distinct physiological properties that suggest that these cell types represent inhibitory interneurons and excitatory projection neurons of the nidopallium. Moreover, these cell classes show distinct and characteristic numerosity-tuning functions in adjacent neurons. The tuning patterns of adjacent NS and BS cell classes, in particular of cell pairs that have been shown to be functionally connected, suggest distinct roles of NS and BS in pallial microcircuits for sculpturing abstract neuronal tuning during stimulus presentation and working memory in a behaviorally relevant way.

The width of the action potential and other physiological-response properties suggested that BS and NS, on average, represent projection neurons and interneurons, respectively^32,44^. This conclusion is based on studies in songbirds in which electrophysiology and histology were combined to identify the types of recorded neurons. Using intracellular recordings from song nuclei of songbirds, narrow waveforms and broad waveforms of spikes could be directly assigned to interneurons and projection neurons, respectively^29,30^. Shallower-rising slopes and slower action potentials in BS relative to NS probably have to do with different membrane kinetics. Intracellular studies in the mammalian neocortex have found that repolarization of the membrane potential following a spike is slower among BS neurons^45–47^. This translates into a broader and shallower peak relative to the trough following the initial trough for extracellular spikes^37^. Similar processes seem to differentiate avian BS and NS.

In addition to waveform parameters, we confirm firing-rate differences of putative interneurons and projection neurons reported in the literature for both birds and mammals. First, the proportions of 81% BS and 19% NS in the NCL correspond to those previously reported in the bird’s brain^32,33^ and in mammals. Intracellular recording and morphological studies in the mammalian neocortex have estimated that roughly 70–80% of neocortical neurons are excitatory pyramidal-projection neurons^26,36,45^.

Second, we found that NS exhibited a higher baseline activity than BS. While NS neurons spiked roughly 6 times per second on average, BS neurons spiked three times per second on average. Our findings are in line with previous studies on avian neurons^32,44^, but also neocortical neurons in mammals^48–51^.

Third, we found much stronger stimulus-evoked responses in NS compared with BS. This property has previously been demonstrated for NS and BS in the song system^44^. A similar effect has been reported in the mammalian neocortex^45,48,49,52–54^. This effect might have been achieved by stronger and faster subthreshold-excitatory postsynaptic potentials (EPSPs) in NS cells than in BS cells^55^. It suggests that putative interneurons elicit inhibitory postsynaptic potentials (IPSPs) in projection cells to restrict spike generation, in a manner consistent of feedforward inhibition thought to operate in disynaptic neural circuits. In addition, putative interneurons exhibited ramping activity right before the stimulus onset, while also showing a much larger increase of firing rates after stimulus onset than BS cells. This may reflect the propensity of NS cells to integrate EPSPs quickly, followed by intense temporal summation of EPSPs in order to provide fast and precise disynaptic inhibitory control.

Fourth, we found faster response latencies to stimulus onset in NS. This again corresponds to findings in the primate neocortex where putative inhibitory neurons had shorter response latencies compared with putative excitatory neurons^49,56^. One interpretation is that short-latency inhibitory responses are likely to sculpt visual processing in excitatory neurons, which in turn yield a sparser visual representation^56^. These distinct physiological properties of NS and BS neurons strongly suggest two different types of neurons in NCL that putatively belong to the classes of inhibitory interneurons and excitatory projection neurons. Despite all of these functional evidences suggesting that BS and NS in the crow NCL represent projection neurons and interneurons, a limitation of the current study is the missing direct anatomical identification of putative projection and inhibitory interneurons in the crow NCL. Future studies need to combine physiology and histological staining of recorded NCL neurons to directly resolve this issue.

The different physiological properties of NS and BS suggest that these cell classes may have different functions in neuronal circuits. We therefore tested the roles of NS and BS in a cognitive task involving abstract categorization and memorization of numerical quantity that is known to be represented in the NCL.

Neurons in the NCL encode different quantities by tuning to preferred numerosities. Therefore, we first compared the tuning properties of these classes of NCL neurons. It turned out that BS shows a sharper tuning curve than NS. This means that putative projection neurons were more selective for numerical categories than putative inhibitory interneurons.

This finding matches numerosity tuning of BS and NS in the primate PFC^49,57^. Beyond numerosities, many studies have reported general broader tuning of putative interneurons over putative projection neurons in the pallium, both in the monkey PFC^39,40,49,58^ and the avian auditory cortex^33^. The high responsiveness of putative interneurons to a broad range of stimuli resulting in broad tuning is thought to be an important component of local microcircuits; it helps interneurons to inhibit nonoptimal responses of projection cells, thus increasing projection neurons’ selectivity.

The notion that interneurons shape the tuning of projection neurons gains further support by an additional important finding: adjacent BS neurons recorded at the same electrode tip showed similar tuning profiles and preferences, whereas neighboring NS and BS tended to show inverse tuning relative to one another. Exactly the same effects were reported for putative interneurons and pyramidal cells encoding numerosity in monkey PFC^49^. This “sculpturing function” of NS on BS is supported by the finding that NS cells showed shorter response latencies, making them ideal candidates for exerting rapid inhibitory feedforward effects on putative projection neurons.

These suggestions of course only apply if different cell types are functionally connected. By exploring temporal firing correlations between neuron pairs simultaneously recorded at the same electrode tip, we were able to establish functional connectivity. Indeed, adjacent NS and BS tended to show a negative correlation of the temporal discharge alongside inverse tuning to numerosity. In those NS–BS pairs, the discharge of one neuron was significantly inhibited by the discharge of its adjacent neuron, the mechanism required to sharpen tuning curves of projection neurons by interneurons. In contrast, neighboring and functionally connected BS-cell pairs exhibited more often positive correlated spike timing, suggesting that they were synchronously excited, perhaps via common input. Overall, the combination of tuning properties and spike timing in functionally connected NS and BS suggests their distinct roles in microcircuits of the crow NCL.

In the current study, we found evidence for putative interneurons in the corvid NCL inhibiting possible projection cells with inverse tuning properties. Representations of abstract categories like quantity categories might first be processed by broadly tuned putative interneurons enabled by their fast-response kinetics and subsequently conveyed to putative projection neurons. In this feedforward mechanism, suboptimal output of putative projection cells, i.e., spiking activity as a response to nonpreferred stimuli, can be inhibited by faster-acting interneurons, which are specifically tuned to the nonpreferred stimuli. In line with this feedforward-inhibition model, it has been argued previously that lateral inhibition is primarily present in cell pairs with dissimilar tuning profiles^26,39^. Our results confirmed this observation in the avian nidopallium. Conversely, spiking activity of putative projection neurons to preferred stimuli is not diminished, since possible inhibitory interneurons are inversely tuned and therefore get activated less often by the preferred stimuli of the adjacent projection cells. As a result, the shoulders of a projection cell’s tuning curve are lowered by this inhibition, since the nonpreferred stimuli are represented at the flanks of the tuning function, ultimately sharpening the tuning curve.

In conclusion, our results suggest that abstract-category tuning in the avian NCL during sensory and working-memory processes is governed by feedforward mechanism. The same mechanism has been proposed for tuning to stimulus properties in mammalian sensory neocortices^59,60^ and the mammalian high-level association neocortex, the PFC^26,49^. As the mammalian and bird telencephalons evolved very distinctly and in parallel from a last common stem amniote that lived over 320 Mio. years ago, two evolutionary scenarios may explain this commonality. One scenario posits that the respective pallial microcircuits have been conserved over this long time of evolution to become part of the avian nidopallium and the mammalian neocortex. Alternatively, the reported feedforward mechanism, together with its building blocks, the excitatory projection neurons, and the inhibitory interneurons, has been reinvented independently in the avian and mammalian lineages to serve similar computational functions to shape and sharpen tuning. The latter hypothesis is supported by the finding that pallial circuits in birds engage an entirely separate classes of excitatory and inhibitory neurons that have no counterpart in the mammalian neocortex^10^. Based on circuitries inherited from common ancestry, macro- and microscale networks and areas reveal that both birds and mammals evolved similar neural and computational properties in parallel and partly independently based on convergent evolutionary forces^4,61^.

## Methods

### Subjects

Three male crows (*Corvus corone*) from the institute’s facilities were used in these experiments. The crows were housed in social groups in spacious indoor aviaries^62^. They were maintained on a controlled feeding protocol during the sessions, i.e., they earned food during daily tests; if necessary, food was supplemented after the sessions. All animal preparations and procedures fully complied with the NIH Guide for Care and Use of Laboratory Animals and were approved by the local national authority (Regierungspräsidium Tübingen, Germany).

### Experimental setup

The crows sat on a wooden perch inside an operant conditioning chamber. Stimuli were presented on a touchscreen monitor. Two different monitors with equivalent specifications were used across studies, a 3 M Microtouch monitor (15 inches, 60 Hz refresh rate) in Ditz and Nieder^21,22^, and an ART-Development PS-150 monitor (15 inches, 60 Hz refresh rate) in Ditz and Nieder^23^. The program CORTEX (National Institute of Mental Health) presented the stimuli on this screen and stored behavioral data. Neuronal data were recorded using a PLEXON system (Plexon Inc.). Reward was given by a custom-built automated feeder below the screen, which delivered either mealworms (Tenebrio molitor larvae) or birdseed pellets upon correctly completed trials. An infrared-light barrier activated by a reflector attached to the birds’ heads ensured a stable head position by registering when the bird was positioned in front and facing the screen.

### Behavioral protocol

The crows were trained on a delayed match-to-sample (DMS) task displaying dots in different numerosities (Fig. 1a). In the initial training protocol, the crows were first trained to perform a DMS with colored squares as discriminative stimuli. The squares were then replaced by different numbers of dots of the same size. Once the crows performed the DMS with same-size dot arrays at high performance (>75%), the displays were controlled for non-numerical cues and the crows further shaped to perform the final task.

Small numerosities (one to four and one to five) and large numerosities (number space between one and thirty) were tested. The large-numerosity space was covered by seven numerosities: 1, 2, 4, 7, 12, 20, and 30. To initiate a trial, the crows had to move their head into an infrared-light barrier when a go-stimulus (O, 3 × 4 mm) was shown on the screen. Throughout the trial, they had to keep their heads in a stable position; a head movement before the onset of the response period was detected by the light barrier and the trial was aborted. As soon as the crows kept their heads at the defined location, the go-stimulus turned off and a 600-ms or 500-ms presample with a gray-background circle on the screen was shown, followed by a display showing the sample numerosity. The sample stimulus disappeared after 800 ms and the crows had to memorize the sample for 1000 ms while only a gray-background circle was visible (delay phase). In the following 800-ms test phase, the first test stimulus was a match in 50% of the cases, i.e., it showed the same number of dots as the sample numerosity, but differed in appearance. The birds indicated a match by either pecking on the screen or by moving their heads out of the light barrier. In the other 50% of the cases, the first test stimulus was a nonmatch showing more or fewer dots than the sample display. The crows had to refrain from responding and had to wait the 800 ms, until a second test stimulus appeared. The second test stimulus was always a match, which the crows had to indicate with a peck on the screen or a head movement. Correct trials were rewarded with food via the automated feeder, whereas error trials led to a timeout of 3 s. If no response occurred within 1600 ms, the trial was dismissed. All relevant task parameters (match/nonmatch, numerosity, and standard vs. control) were balanced.

### Stimuli

Three different stimuli sets were used. They consisted of dot displays with varying numbers of dots: 1–4 dots, 1–5 dots and 1–30 dots covered by the numerosities 1, 2, 4, 7, 12, 20, and 30. The black dots with a diameter range 0.4–2.8° of visual angle were displayed on a gray-background circle with a diameter of 10° visual angle. To prevent crows from performing visual-pattern matching, sample and test images were never identical. Furthermore, each numerosity was displayed in dot displays with random positions and randomly varying sizes. All displays were exchanged for each training session with newly pseudorandomly generated displays by a custom-written MATLAB script in order to prevent the crows from memorizing the visual patterns to solve the task.

To ensure that the crows would not use low-level features, like visuospatial cues, to solve the task, but instead the discrete quantity displayed, control stimuli were introduced in addition to the standard stimuli (Fig. 1b). The standard-trial stimuli showed nonoverlapping dots of varying size in varying locations. In the control condition, the dot area, defined as the cumulative surface of all dots on a numerosity display, and the dot density, defined as the average distance between the centers of all dots on a numerosity display, was equal across all numerosities. In one of the three stimuli sets, the total circumference of all dots on a display was equated. In addition, a linear control was used, i.e., two or more dots formed a line. These two controls were not used in the other stimuli sets, because it became apparent that these visual parameters only had a negligible effect on NCL neurons. Standard and control trials were shown with equal proportions in each session and randomly alternated.

### Surgery and recordings

All surgeries were performed while the animals were under general anesthesia. Crows were anesthetized with a ketamine/xylazine mixture as described in Ditz and Nieder^21^. After the surgery, the crows received analgesics^21^. The head was placed in the stereotaxic holder that was customized for crows with the anterior fixation point (i.e., beak-bar position) 45° below the horizontal axis of the instrument. Using stereotaxic coordinates (center of craniotomy: anterior–posterior +5 mm relative to interaural (ear bars) as zero; medial–lateral 13 mm relative to midline), we chronically implanted up to four microdrives with several electrodes each into the left or right hemispheres, a connector for the head stage, and a small head post to hold the reflector for the light barrier. Glass-coated tungsten microelectrodes with 2 MΩ impedance (Alpha Omega) were used. The electrodes targeted the corvid NCL, which is characterized by dopaminergic cells^63–65^. Each recording session started with adjusting the electrodes, until a proper neuronal signal (of at least 3:1 signal to noise) was detected on at least one channel (see also Fig. 4a, b in Veit and Nieder^63^, for an example recording trace). Neurons were not preselected in the involvement of the task. Each microdrive had a range of ~6 mm, which was exploited to record from the NCL across different depths over a period of several weeks.

Every session, the birds were placed in the recording setup and a head stage containing an amplifier was plugged into the connector implanted on the bird’s head and connected to a second amplifier/filter and the Plexon MAP box outside of the setup by a cable above and behind the bird’s head (all components by Plexon). Signal amplification, filtering, and digitizing of spike waveforms was performed using the Plexon system. Spectral filtering of recordings was accomplished by a combined preamplifier filter (150 Hz–8kHz, 1-pole low-cut, 3-pole high-cut) and main filter (250 Hz, 2-pole, low-cut filter). Amplitude amplifications were set individually for different channels in the range of ca. 20,000x gain. Spike waveforms were sampled at a frequency of 40 kHz (one entry every 25 µs) for a duration of 800 µs, resulting in a 32-element vector. Plexon’s Offline Sorter was used to manually offline sort spikes into single-unit waveforms by applying mainly principal-component analysis. We recorded one neuron on 491 sites, two neurons on 201 sites, three neurons at 61 sites, and four or more neurons on 9 sites.

We verified the NCL and the location of the electrodes in NCL (according to the implantation coordinates provided above). We immunohistochemically stained for tyrosine hydroxylase to identify dopaminergic cells, which characterize the NCL^65^ (see Fig. 3 in Veit and Nieder^63^). Next, we traced electrode tracks to confirm that recording locations were within NCL (see Fig. 3a, b in Veit et al.^66^). We targeted the medial part of the nidopallium caudolaterale (mNCL according to Sen et al.^67^).

### Selection criteria

Only neurons, which were recorded for at least four repetitions of each sample numerosity per protocol type (standard and control), and which had a firing rate of at least 0.5 Hz during sample and delay period, were included in the analysis. Furthermore, the mean waveform of each neuron had to fulfill the following selection criteria: the minimum of the waveform had to occur between 150 and 400 µs after reaching the initial threshold. The maximum had to occur only after 300 µs after the initial amplitude threshold was reached by the waveform, so that only mean waveforms of neurons with a downward voltage deflection followed by an upward deflection with a clear peak were included. Every other neuron was excluded from further analysis. In total, 33 neurons were excluded, leaving 1136 neurons from the initially 1169 recorded units for further analysis.

### Classification of narrow- and broad-spiking neurons

In a preprocessing step, waveforms were normalized by the difference between their peak and trough values and aligned by their minimum to ensure that the spike width was standardized for each waveform, and for a classifier, nonessential features of the waveform (i.e., amplitude and time of minimum) were disregarded. In a first step, the difference between the minimum and maximum amplitude was calculated (the magnitude of the amplitude). In a second step, the original waveform was divided by the magnitude of the amplitude. Thus, the normalized amplitude of the waveform equals 1. Then, the waveforms are aligned to the occurrence of the minimum.

Spike widths were then used by a linear classifier, the k-means algorithm (k = 2, squared Euclidean distance), to categorize single units as narrow- or broad-spiking cells^37^. The cluster with the smaller mean spike width was defined as the population of narrow-spiking neurons and consisted of 218 cells, while the second cluster, consisting of 918 cells, had a larger mean spike width and therefore was defined as the population of broad-spiking neurons. To evaluate the classification, several physiological properties of NS and BS neurons, such as rising slopes of waveforms, baseline activity, stimulus-evoked firing rates, cell-type proportions, and visual-response latency, were analyzed.

### Numerosity neurons

Neurons were defined as numerosity neurons, if they were activated by the numerosity shown in dot displays rather than their low-level visual features. To find neurons, which were solely activated by abstract numerosities, a two-factor ANOVA was separately calculated based on the discharge rates in the sample and the delay phase. The two main factors of the ANOVA were numerosity (1–4, 1–5, and 1–30) and stimulus condition (standard and control). Cells were considered numerosity-selective only if they showed a significant main effect of numerosity, but no significant stimulus condition or interaction effect in either the sample or delay period. To derive the baseline activity, the mean discharge rate during the 250 ms prior to the sample period was analyzed.

### Visual-response latency analysis

The visual-response latencies of single neurons were assessed by examining the spike density histogram of each neuron. The histograms had a resolution of 1 ms but were smoothed by a sliding window with a kernel bin width of 10 ms. A 200 ms time window before stimulus onset was used as a baseline period. If five consecutive time bins after stimulus onset reached a value higher than the maximum of the baseline period, response latency was defined by the first of these time bins. Latencies smaller than 50 ms were labeled as false positives and eventually disregarded. Equally, neurons with latencies longer than 250 ms, or those that never fulfilled the criteria, were precluded.

### Cross-correlation between tuning curves of neuron pairs

To assess the difference in numerosity tuning of adjacent neurons, we obtained their tuning curves and calculated the cross-correlation coefficient (CC_Tuning_). The tuning curves are defined as vectors ${t}_{{{{{{\rm{neuron}}}}}}1}$ and ${t}_{{{{{{\rm{neuron}}}}}}2}$ with entries ${t}_{n}$ being the mean firing rate in Hz of numerosity $n$. The CC_Tuning_ then takes the discharge rates of each numerosity of each neuron pair, makes them scale-invariant by subtracting the means ${\bar{t}}_{{{{{{\rm{neuron}}}}}}1}$ and $\bar{t}$_neuron2_ of each discharge rate and enables comparison across cell pairs with a dimensional reduction to a scalar. The normalized cross correlation coefficient CC_Tuning_ was calculated as follows:

1 $${{{{{\rm{CC}}}}}}_{{{{{\rm{Tuning}}}}}}=\frac{\mathop{\sum }\nolimits_{n=1}^{{n}_{{\max }}}\left({t}_{{{{{{\rm{neuron}}}}}}1}\left(n\right)-{\bar{t}}_{{{{{{\rm{neuron}}}}}}1}\right)\times \left({t}_{{{{{{\rm{neuron}}}}}}2}\left(n\right)-{\bar{t}}_{{{{{{\rm{neuron}}}}}}2}\right)}{\sqrt{\mathop{\sum }\nolimits_{n=1}^{{n}_{{\max }}}{\left({t}_{{{{{{\rm{neuron}}}}}}1}\left(n\right)-{\bar{t}}_{{{{{{\rm{neuron}}}}}}1}\right)}^{2}}\times \sqrt{\mathop{\sum }\nolimits_{n=1}^{{n}_{{\max }}}{\left({t}_{{{{{{\rm{neuron}}}}}}2}\left(n\right)-{\bar{t}}_{{{{{{\rm{neuron}}}}}}2}\right)}^{2}}}$$

where ${t}_{{{{{{\rm{neuron}}}}}}1}$ is the tuning function for neuron 1, ${t}_{{{{{\rm{neuron}}}}}2}$ is the tuning function for neuron 2, ${\bar{t}}_{{{{{\rm{neuron}}}}}1}$ and $\bar{t}$_neuron2_ are the means of the tuning functions, and $n\in \{{{{{\mathrm{1,2}}}}},\ldots ,{n}_{{\max }}\}$ with ${n}_{{\max }}\in \left\{{{{{\mathrm{4,5,30}}}}}\right\}$ being the numerosity. The means were calculated as follows:

2 $${\bar{t}}_{{{{{{\rm{neuron}}}}}}1}=\frac{1}{{n}_{{\max }}}\mathop{\sum }\limits_{n=1}^{{n}_{{\max }}}{t}_{{{{{{\rm{neuron}}}}}}1}(n){{;}}\,{\bar{t}}_{{{{{{\rm{neuron}}}}}}2}=\frac{1}{{n}_{{\max }}}\mathop{\sum }\limits_{n=1}^{{n}_{{\max }}}{t}_{{{{{{\rm{neuron}}}}}}2}(n)$$

A positive CC_Tuning_ value indicates that the corresponding cell pair holds two similar numerosity tunings while a CC_Tuning_ value of 1 describes identical tuning curves. On the other hand, negative CC_Tuning_ values suggest an inverse tuning of both cells with a CC_Tuning_ value of −1 describing mirrored tuning functions.

Only neurons that were significantly tuned to numerosity (as tested by the two-way ANOVA), and in addition had been recorded at the same electrode, were included in the cross correlation analysis.

### Cross-correlation between spike trains of neuron pairs

The temporal cross correlation (CC_Timing)_ is a measurement of correlation between single spike events of two neurons^68^. To calculate the temporal cross-correlation, the whole trial of each condition was taken into account, and the time period in which both neurons of a given neuron pair were simultaneously active at a given electrode was analyzed. A custom-made MATLAB algorithm calculated the temporal cross-correlation values for each neuron pair as follows: of a given neuron pair, the temporal delays of each spike of neuron 1 to each spike of neuron 2 were calculated and plotted in a histogram with a bin width of 1 ms. This procedure was repeated for all spikes and all trials. All temporal delays were then summed up in a cross-coincidence histogram (CCH).

Subsequently, a shift predictor was subtracted from the CCH to control for simple rate covariation. The shift predictor was calculated by correlating trials of neuron 1 with subsequent trails of neuron 2 and the last trial of neuron 1 was correlated with the first one of neuron 2. The shift predictor can therefore be interpreted as an estimate for correlogram features induced by an influence repeating itself in all trials (typically, the stimulus).

To determine which neurons had significant cross-correlation peaks, we calculated the CCH from time-shuffled data for each cell pair and compared the peak values for the observed and shuffled data sets. Time-shuffling the data (i.e., each spike was moved in time, destroying local correlations, but the firing rate was kept the same) a hundred times for every cell pair produced a shuffled distribution of cross-correlation values. Significant cross-correlation peaks were detected by a permutation test; peak values in the real CCH that deviated from the confidence interval of 95% of the shuffled distribution of values were considered significant (*p* < 0.05). We looked at peaks of the CCHs within ±50 ms time. We considered those peaks as significant, which exceeded the mean peak size of 95% of the time-shuffled data^69^.

### Statistics and reproducibility

Statistical tests were calculated in MATLAB. Neurons’ numerosity selectivity was tested using a two-way analysis of variation (ANOVA). Bimodal distributions were tested Hartigan’s dip test for unimodality. Independent firing-rate distributions were compared using Mann–Whitney U-tests. Neuron frequencies were compared with a *χ*^2^-test. Significant cross-correlation peaks were detected by a permutation test in relation to confidence interval of 95% of the shuffled distribution.

### Reporting summary

Further information on research design is available in the Nature Research Reporting Summary linked to this article.

## Supplementary information

Description of Additional Supplementary Files

Supplementary Data 1

Reporting Summary

## Data Availability

Source data underlying the main figures are presented in Supplementary Data 1. All other data are available from the corresponding author on reasonable request.

## Code availability

Matlab code and data to reproduce figures are publicly available at https://data.mendeley.com/datasets/7kmtz7kj4c/1.
