# Supplementary material for: Cell-type specific pallial circuits shape categorical tuning responses in the crow telencephalon
Source: Commun Biol. 2022 Mar 25;5:269. doi: 10.1038/s42003-022-03208-z (PMC8956685; doi:10.1038/s42003-022-03208-z)
Supplement: Supplementary file 1 — Description of Additional Supplementary Files [file 42003_2022_3208_MOESM1_ESM.pdf]

Description of Additional Supplementary Files

File name: Supplementary Data 1
Description: Source data underlying main figures.
